# "The Hospital" to Its Readers

**Published:** 1897-04-03

**Authors:** 


### "The Hospital" to Its Readers.

In commencing a new volume it is but appropriate that a word should be said to our readers as to the aims and objects of The Hospital. A moment's inspection of our pages will suffice to show that in several points we differ from ordinary medical journals conducted on the old lines. The *Lancet* and the *British Medical Journal* honourably fulfil their important mission. With the large number of pages at their disposal they are able to insert in full many ponderous communications which are of the utmost value, and it may be conceived that if any means were provided by which the chaff might be winnowed out from the wheat no better library could be possessed by a medical man than a long regiment of these fat volumes. Unfortunately, they are fat; and the very fact that th ir great value ies in the original papers buried in their depths leads many men to bind rather than to read them.

We hope that the majority of our readers take the heavy journals also, but we none the less think that The Hospital, which, in its first half-dozen pages, gives a weekly record of the gist of what is going on in the world of medicine, will be found by all medical men, as we know it is by many, to be a welcome *résumé* of medical progress. It is light, it is cheap, it is readable, both as regards its matter and its typography; every effort is made to render it thoroughly reliable both from a "news" and a scientific point of view; it may be thrown away when done with, or, if the reader is of a more careful bent, it may be bound, to form a goodly volume and most useful record of the work and progress of the year; and, lastly, it contains within it a separately bound section—"The Nursing Mirror"—which is always a welcome gift to any nurse with whom the medical reader may for the moment be associated, in whatever quarter of the globe he practises.

So far as The Hospital proper is concerned, putting the "Mirror" for the moment on one side, it is addressed primarily to those who have to deal with the treatment of disease in hospitals, asylums, workhouses, and infirmaries; honorary physicians and surgeons, house surgeons, superintendents, and medical officers. It need not be said, however, that from the special prominence which it gives to all that concerns hospital management, The Hospital is much read by secretaries, stewards, matrons, and even by members of committees—by all, in fact, who are interested in managing hospitals. In regard to this side of our work, a word of explanation, and even a word of protest against calumny, may not be out of place. The very fact of our being popular among those whom doctors are fond of calling the "lay element" in hospital committees (on account of our containing information on hospital affairs which they cannot readily obtain elsewhere), has led certain narrow-minded individuals to pretend that The Hospital is not a medical journal. In regard to this it may properly be asked what rules are adopted by, and what restrictions are placed upon, papers which are universally recognised as medical journals, and to which the most rigid disciplinarians, as regards medical ethics, do not hesitate to send their contributions; we refer, of course, to the *Lancet* and the *British Medical Journal!* In our view, both these are most admirable papers. But is it true that they are read by the medical profession alone? Are they not sold on bookstalls? are they not to be found on club and hotel tables? are they not taken at free libraries? are they not subscribed for by mechanics' institutes, and gloated over by the educated but unwashed youth of the period? We know that they are. Perhaps it will be said that these are accidents beyond the control of any newspaper manager, and that, although they may be read by all sorts of people, these papers are addressed to the medical profession alone. Never was a greater untruth. What about the agitations raised in regard to workhouses, pauper schools, water supply, oysters, pilgrimages, &c.; can it b pretended for one moment that the articles on these subjects which illuminate the pages of the *Journal* from time to time are addressed to the profession alone? A little time ago the *Lancet* published a most useful supplement containing complete specifications for the sanitary fittings of a house, so far as the plumber was concerned; was this addressed to the profession alone? The suggestion is childish. From its commencement the *Lancet* has been powerful just in proportion as it has addressed the public in the name of the profession, and the same may be said in regard to the *British Medical Journal.* Both these papers address themselves to the great outside public in the name of medicine, and so do we. The Hospital is written by medical men for medical men, and in that narrow sense is a technical medical newspaper. But it also puts into words—"voices," as the saying is—the feelings and opinions of the medical profession on those great questions of the day on which medicine may be expected to speak with authority. In doing so it certainly aims at the ear of the public, and so does every medical journal that is worthy of being read.

